# Granulomatosis With Polyangiitis (Wegener's Granulomatosis) Complicated by Pericarditis: Our Experience of Two Cases and Comparative Review of Literature

**DOI:** 10.1016/j.case.2020.11.008

**Published:** 2021-01-26

**Authors:** Taha Ahmed, Dane Meredith, Allan L. Klein

**Affiliations:** Center for the Diagnosis and Treatment of Pericardial Diseases, Heart, Vascular and Thoracic Institute, Cleveland Clinic, Cleveland, Ohio

**Keywords:** Granulomatosis with polyangiitis, Acute pericarditis, Recurrent pericarditis, Anakinra, Corticosteroids, Cardiac magnetic resonance imaging

## Abstract

• GPA is a systemic necrotizing vasculitis of medium and small vessels.
• GPA classically involves the upper and lower respiratory tracts and the kidneys.
• Pericarditis is a common cardiac manifestation, but RP is rarely described.
• A systematic literature search yielded 13 cases of acute pericarditis secondary to GPA, which are analyzed in this review.
• The potential role of Anakinra for debilitating RP secondary to GPA is described.

## Graphical abstract

**Figure:**
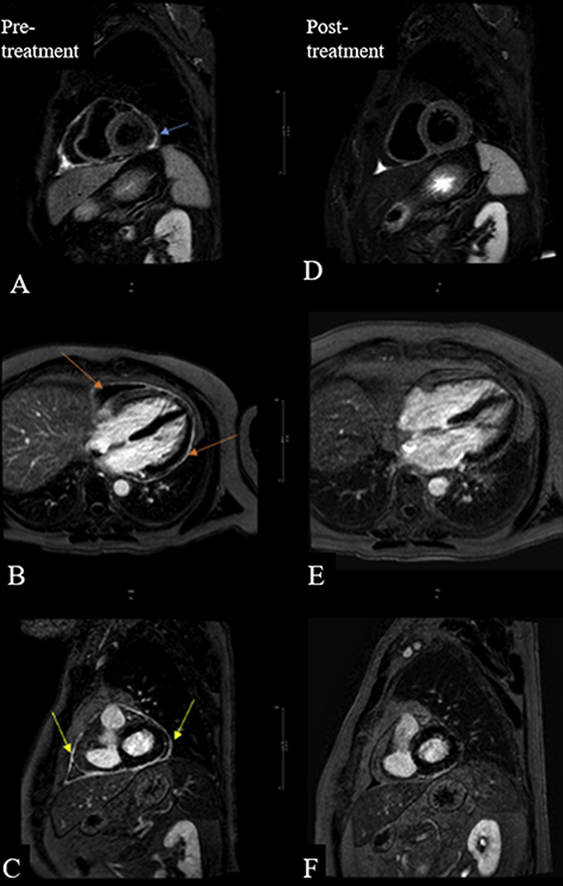

## Introduction

Granulomatosis with polyangiitis (GPA; formerly known as Wegener's granulomatosis) is a disease characterized by necrotizing granulomatous vasculitis involving the upper and lower respiratory tracts and the kidneys.^1^ Cardiac involvement is reported in 6%-44% of cases, and pericarditis is the most common cardiac manifestation.^2^^,^^3^ The pathophysiology is postulated to be necrotizing vasculitis secondary to granulomatous infiltrates.^4^^,^^5^ Prognosis is same as for other forms of pericarditis, and in cases where constrictive pericarditis develop, surgical intervention is associated with good outcome.^3^^,^^6^^,^^7^

Herein, we report two cases of pericarditis secondary to GPA. Furthermore, the systematic review of literature outlines our current understanding of the epidemiology, clinical presentations, diagnostic modalities, clinical course, and outcomes of pericardial diseases in GPA.^1^, ^2^, ^3^, ^4^, ^5^, ^6^, ^7^, ^8^, ^9^, ^10^

## Case Presentation

### Patient 1

A 44-year-old woman, presented to the Emergency Department (ED) from her dialysis center with pleuritic chest pain, shortness of breath, and fever during her dialysis session. The patient has a past medical history significant for GPA diagnosed by renal biopsy revealing crescentic glomerulonephritis and positive p-ANCA/MPO serology. Despite treatment with prednisone and rituximab, the patient progressed to end-stage renal disease. At the time of admission, the patient's physical examination revealed clear lung fields, regular heart rate and rhythm, a pericardial friction rub, flat jugular veins, and no pedal edema.

**Figure 1:**
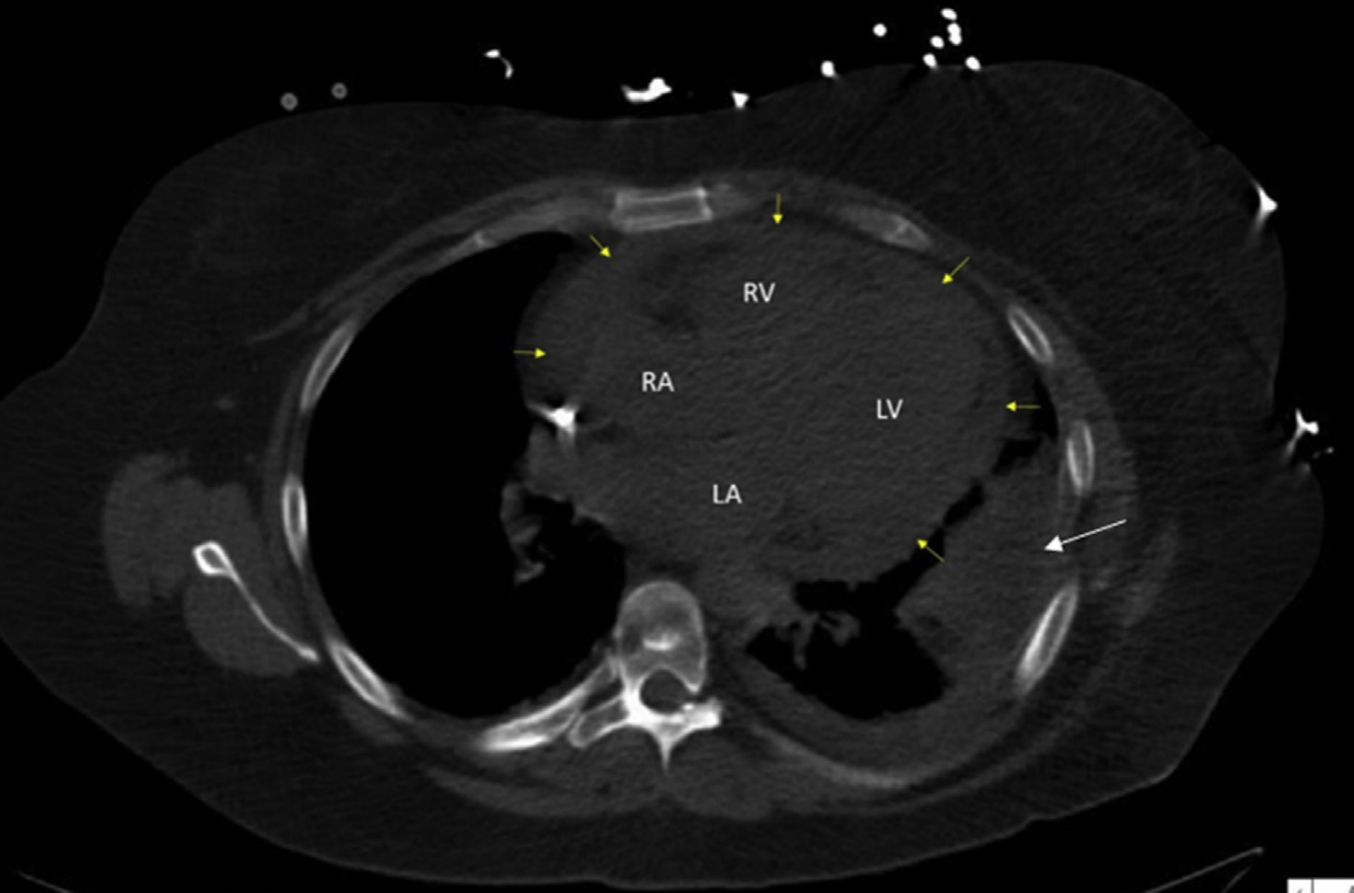
Chest CT scan axial view showing moderate to large circumferential pericardial effusion (*yellow arrows*) and left pelural effusion (*white arrow*). *LA*, Left atrium; *LV* left ventricle; *RA*, right atrium; *RV*, right ventricle.

Initial laboratory studies revealed mild leukocytosis of 11.84 K/uL (normal, 3.7-11.0 K/uL), ESR 88 mm/hour, and CRP 20.3 mg/dL. Blood cultures were negative, but viral swab revealed respiratory syncytial virus. Chest computed tomography (CT) scan on presentation showed a new-onset moderate to large sized circumferential pericardial effusion (Figure 1).

**Figure 2:**
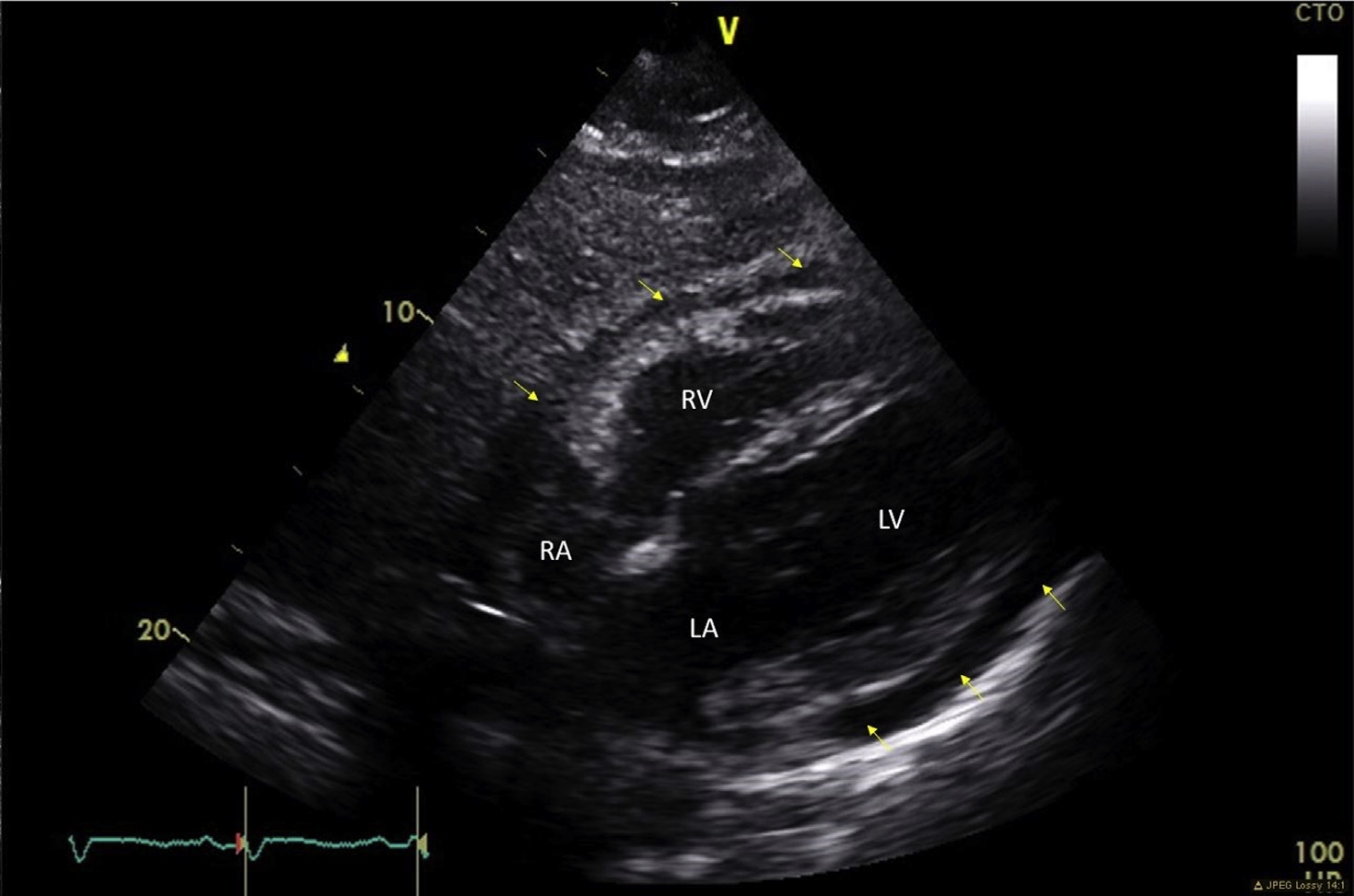
Transthoracic echocardiogram two-dimensional subcostal view of the heart showing moderate size circumferential pericardial effusion without tamponade (*yellow arrows*). *LA*, Left atrium; *LV*, left ventricle; *RA*, right atrium; *RV*, right ventricle.

**Figure 3:**
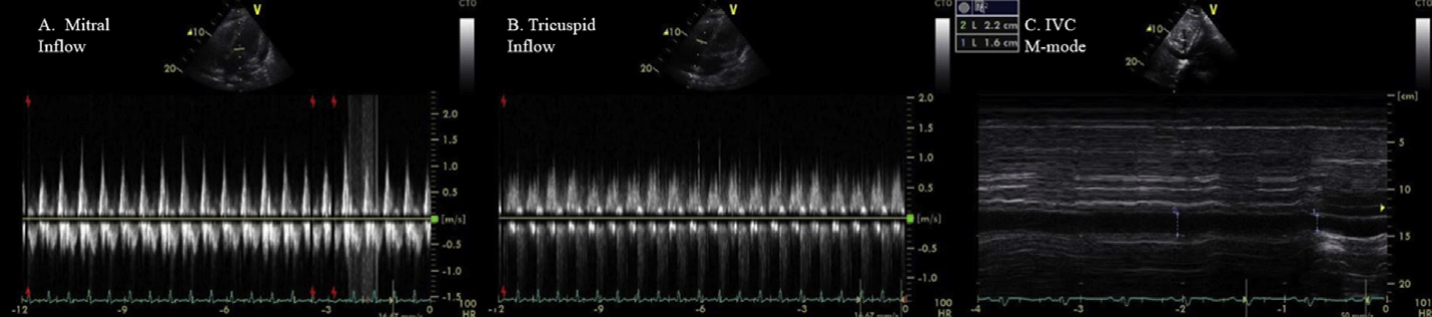
Pulsed-wave-Doppler recordings of **(A)** mitral and **(B)** tricuspid inflow showing mild respiratory variation of mitral and tricuspid inflows in setting of moderate pericardial effusion. **(C)** M-mode echocardiogram of IVC showing dilated IVC (>21 mm diameter) with plethora.

Electrocardiogram showed normal sinus rhythm. Cardiac enzymes were within normal range. Transthoracic echocardiogram (TTE) revealed a moderate sized pericardial effusion and inferior vena cava (IVC) plethora, but no chamber collapse or significant respiratory variation of Doppler inflows to suggest tamponade (Figures 2 and 3; Video 1 available at www.onlinejase.com).

In the setting of classic chest pain, elevated inflammatory markers, and moderate pericardial effusion, the patient was diagnosed with GPA relapse manifesting as acute pericarditis with moderate pericardial effusion with only mild respiratory variation of Doppler inflows (Figure 3A–C; Video 2 available at www.onlinejase.com).

Cardiac magnetic resonance imaging (MRI) was deferred due to renal failure. The patient's prednisone was escalated to 60 mg daily and colchicine 0.6 mg twice daily was added to treat the acute pericarditis in the setting of GPA. Avoidance of physical activity was recommended upon discharge. At 3-month follow-up, the patient reported no symptoms and a trivial pericardial effusion was found on TTE. There was no abnormal respirophasic ventricular septal shift (ventricular interdependence) observed on M-mode echocardiogram. (Video 3 available at www.onlinejase.com).

Continuation of colchicine and a prolonged prednisone taper was advised. At 6-month follow-up, the patient remained asymptomatic. The prednisone was tapered down to 12.5 mg, and colchicine dose was decreased to once daily.

The patient noted recurrence of her pleuritic chest pains as the prednisone was further tapered prompting multiple visits to the ED, subsequently her prednisone dose was increased to 20 mg, resulting in improvement of her symptoms. Inflammatory markers remained normal. Instructions were given for a very slow prednisone taper (5 mg every 2 weeks then subsequently 2.5 mg every 2 weeks), and the patient was approved for renal transplant.

The patient continued to have flares of recurrent pericarditis post–renal transplant and she presented 1.5 years later to our pericardial center with an active flare of her recurrent pericarditis. She was on immunosuppressants tacrolimus and everolimus for her renal transplant. A decision was made to initiate the patient on anakinra (interleukin blocker), in addition to her current medical therapy. Colchicine was stopped due to elevated hepatic enzymes and the patient was continued on quadruple immunosuppressive therapy with tacrolimus, everolimus, prednisone and anakinra, with symptomatic improvement.

**Figure 4:**
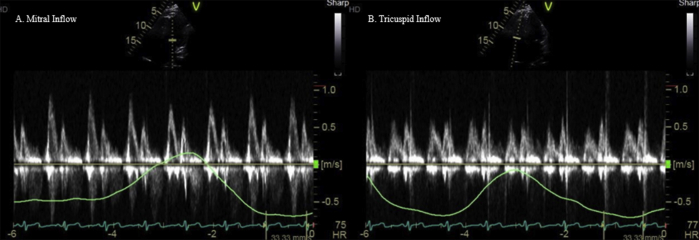
Pulsed-wave-Doppler recordings of **(A)** mitral **(B)** tricuspid inflow velocities, with simultaneous respirometric recording, showing no significant respirophasic variations of mitral and tricuspid inflows.

On most recent follow-up at the pericardial center, 3 years after the index hospitalization, the patient had been asymptomatic with no reported recurrences, ED visits, or hospitalizations in the last 12 months. Transthoracic echocardiogram showed no pericardial effusion or signs of constrictive physiology (Figure 4A and B; Videos 4 and 5 available at www.onlinejase.com). Prednisone were slowly tapered off, given concerns of developing undesirable side effects with chronic steroid therapy, followed by a prolonged anakinra taper with an aim to taper off all medical therapy in a span of next 1 year.

### Patient 2

**Figure 5:**
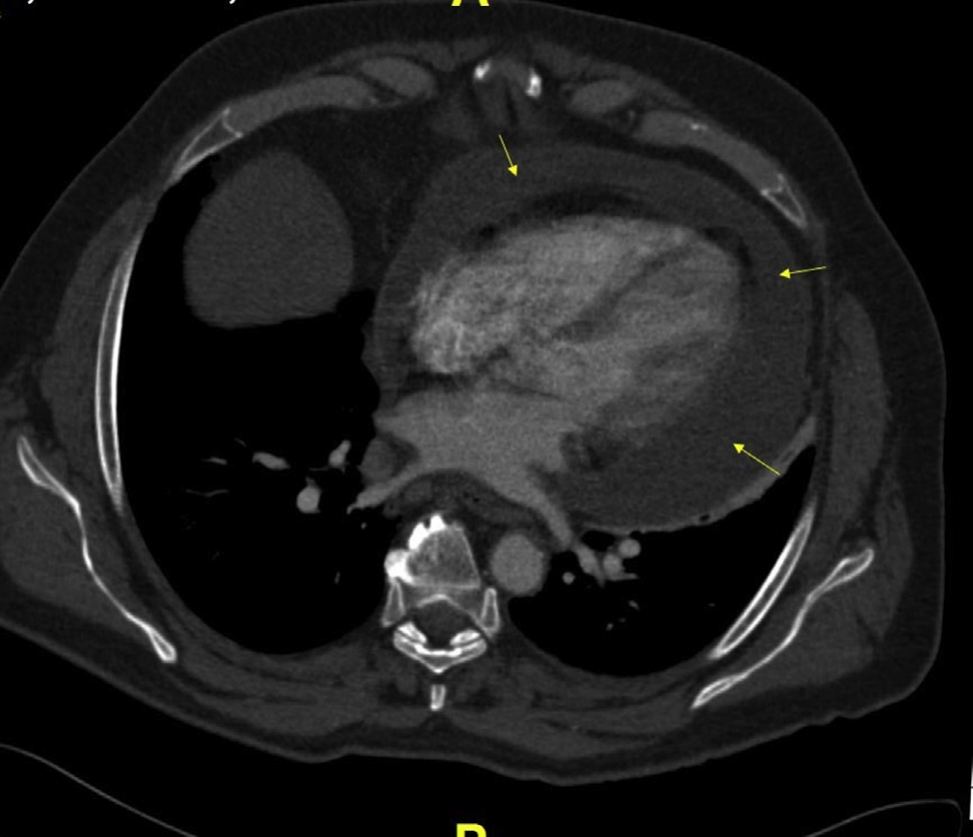
Chest CT scan axial view showing moderate-to-large pericardial effusion (*yellow arrows*).

A 63-year-old man was referred to our clinic for investigation of pericardial effusion. He had recurrent otitis media, resistant to steroid and antibiotic therapy, with hearing loss for last 5 years. The patient was recently at the hospital for worsening shortness of breath, weight loss, night sweats and dry cough for 5 months. An elevated ESR of 71 mm/hour and CRP of 17.5 mg/dL were significant. Chest CT scan showed a moderate-to-large pericardial effusion with flattening of interventricular septum (Figure 5) followed by a TTE showing a moderate-to-large organized circumferential pericardial effusion with no evidence of pericardial tamponade.

The patient underwent pericardiocentesis with 400 mL of fluid drained, analysis of which revealed an exudative effusion with acute and chronic inflammation and no malignant cells. The patient improved symptomatically postprocedure and a repeat TTE revealed a moderate organized pericardial effusion measuring 1.8 cm adjacent to the right atrium and right ventricle. The patient was diagnosed with acute pericarditis and started on colchicine 0.6 mg twice daily and ibuprofen 800 mg thrice daily.

**Figure 6:**
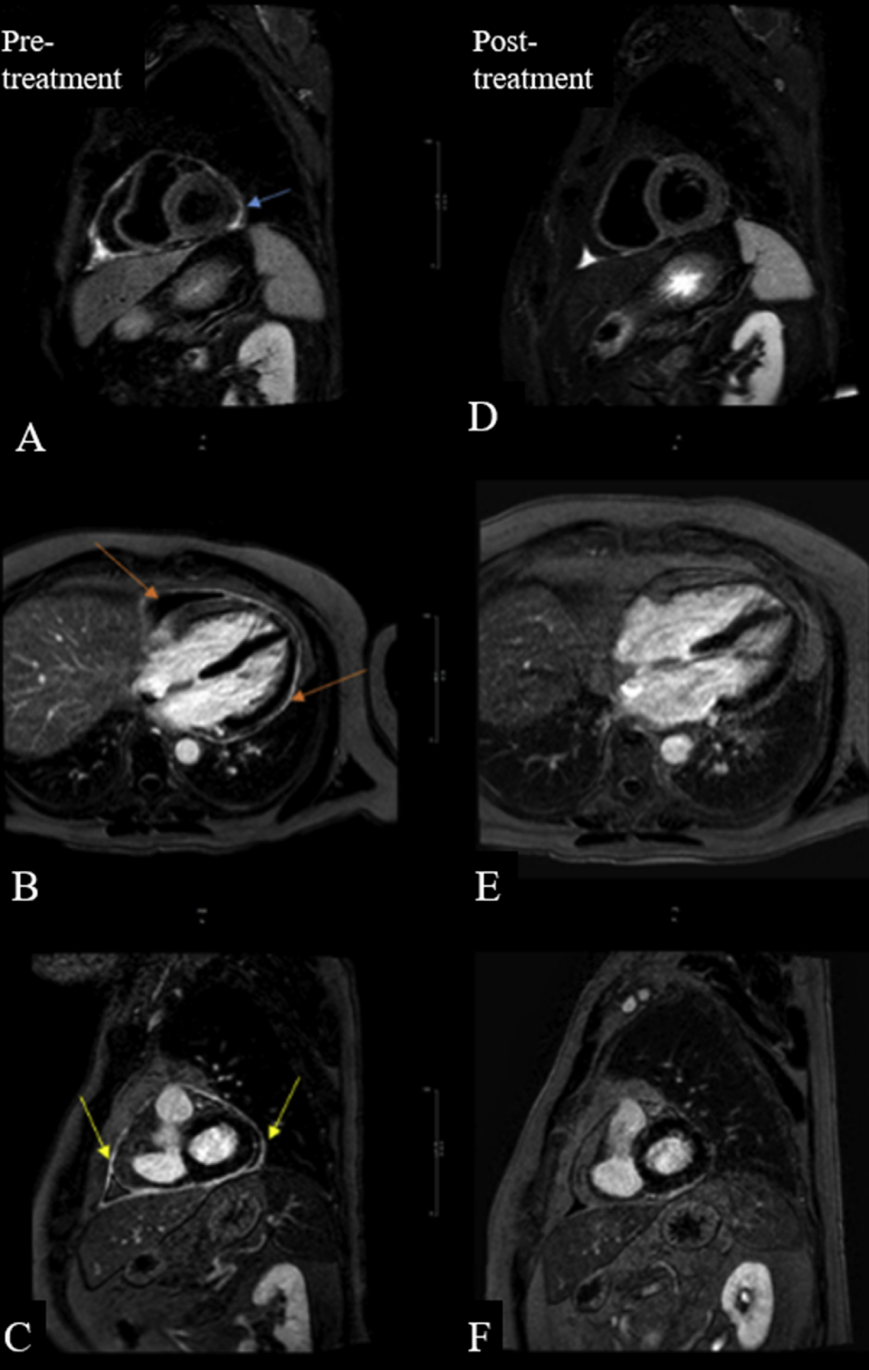
Cardiac MRI with pretreatment images showing **(A)** increased T2 STIR signal intensity indicating acute inflammation, small pericardial effusion, and late gadolinium enhancement in **(B)** four-chamber and **(C)** short-axis views. Cardiac MRI posttreatment images showing no more increased pericardial signal T2 STIR, indicating edema has resolved **(D)**; resolution of late gadolinium enhancement in **(E)** four-chamber **(F)** short-axis views indicating the pericardial inflammation is resolving.

The patient continued to have night sweats and was readmitted 1 month later with fever and proximal muscle weakness. Laboratory workup revealed prominent leukocytosis of 18.68 K/uL, aspartate aminotransferase of 56 U/L (normal, 7-40 U/L), alanine aminotransferase of 71 U/L (normal, 5-50 U/L) and an elevated p-ANCA level (detected by indirect immunofluorescence) of 44 U (normal, 0-20 U). Repeat TTE shows small pericardial effusion. A diagnosis of GPA was made and the patient was started on prednisone 40 mg daily and cyclophosphamide 150 mg daily. A cardiac MRI was performed due to persistent symptoms and poor response to medical therapy. It revealed increased pericardial thickening of 2-3 mm with moderate circumferential enhancement of the pericardium on late gadolinium enhancement sequences, increased signal on T2 edema weighted imaging consistent with active pericarditis without any features of constrictive physiology (Figure 6A–C). The patient showed a dramatic improvement on combination cyclophosphamide and prednisone prolonged taper and a repeat cardiac MRI 4 weeks later showed reduction in pericardial effusion, enhancement and thickening (Figure 6D–F).

**Figure 7:**
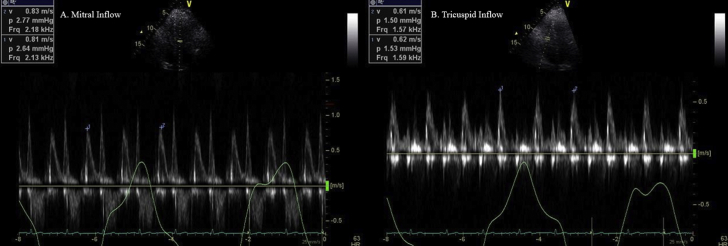
On-treatment Doppler recording of **(A)** mitral and **(B)** tricuspid inflow with a respirometer showing no evidence of constrictive physiology.

The patient continued to follow at the pericardial center. He reported developing fatigue with worsening hearing loss 3 years after the index admission and was started on rituximab infusions. The patient's GPA is currently managed on low-dose prednisone and rituximab infusions with no recurrence of pericarditis (Figure 7A and B; Videos 6–8 available at www.onlinejase.com).

## Discussion

First described in 1931 by Klinger and further characterized by Wegener in 1936, GPA (formerly called Wegener's granulomatosis) most commonly involves the sinuses, lungs and kidneys with necrotizing granulomatous vasculitis.^1^^,^^3^ Cardiac complications can occur in up to 44% of patients with GPA. Pericarditis is the most frequently reported cardiac manifestation of GPA (50% of cases), but myocarditis, endocarditis and conduction system granulomata are also described.^3^ It has been reported that pathologic involvement of the pericardium is found in as many as 50% of patients with GPA at autopsy. Mild subclinical pericardial effusions are found in the majority of patients, and large pericardial effusions and tamponade requiring pericardiocentesis with or without pericardial window is rare.^11^^,^^12^ Pericardial effusions can develop in the absence of severe renal dysfunction, indicating that besides uremia, GPA vasculitis has a direct pathogenic role.^13^ Absence of pathological evidence of granulomata or active vasculitis in pericardial tissue suggests that other inflammatory mechanisms associated with disease exacerbations are involved.^1^^,^^3^^,^^7^ Constrictive pericarditis with GPA is rarely reported.^3^^,^^7^

The etiology of pericardial diseases is diverse and is broadly classified into infectious causes (viral, bacterial, fungal or parasitic) and noninfectious causes (autoimmune, neoplastic, metabolic, traumatic/iatrogenic, drug-related and congenital malformations). Pericarditis related to systemic vasculitis is categorized under autoimmune causes of acute pericarditis.^13^ Pericardial involvement in systemic vasculitides is relatively rare in large-vessel vasculitis while it is more common in medium- and small-vessel vasculitis such as Kawasaki disease, eosinophilic granulomatosis with polyangiitis and GPA (Wegener's granulomatosis).^13^

**Figure 8:**
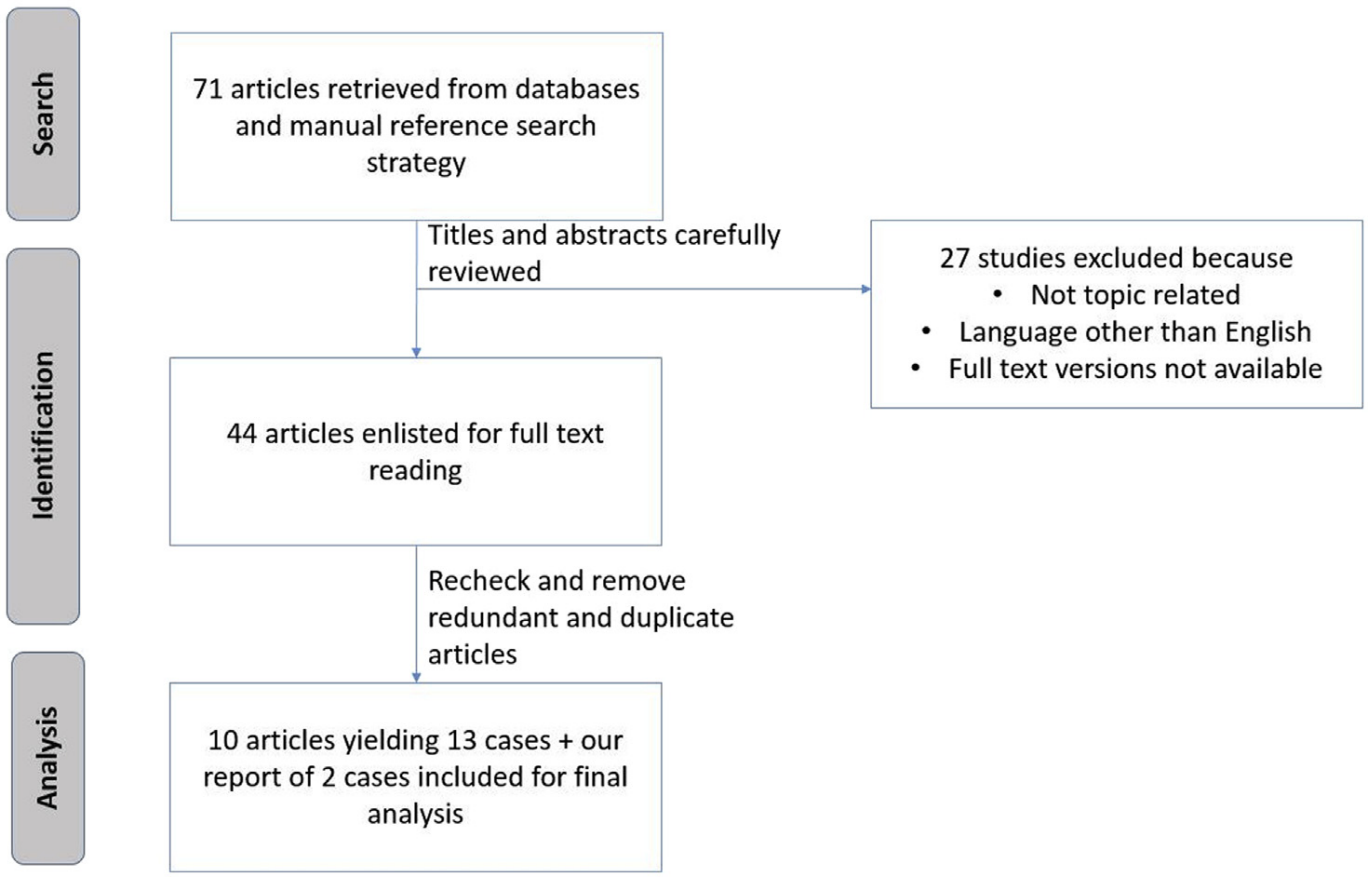
Flow diagram depicting the selection of the articles included in this review.

We systemically searched the published medical literature to retrieve the available case reports for pericardial involvement in GPA/Wegener's granulomatosis (Figure 8). The data of these patients including demographics, clinical features, diagnostic tools, prognosis and outcomes are summarized in Table 1.^1^, ^2^, ^3^, ^4^, ^5^, ^6^, ^7^, ^8^, ^9^, ^10^

**Table 1:** Literature review of case reports of pericardial diseases in GPA (Wegener's granulomatosis)

Author	Publication year	Country	Age/gender	Prior organ involvement	Clinical presentation	Relevant examination findings	Diagnostic EKG and laboratory findings	Multimodality cardiac imaging findings	Pericardial fluid drain/biopsy	Treatment	Clinical course and outcomes	Recurrence
Schiavone *et al* Patient 1^1^	1985	USA	60/M	Nose, lung, kidney	Weight gain, abdominal distension, edema	Pericardial knock	Renal failure	TTE: loculated posterior pericardial effusion, thickened pericardium, abrupt halt in diastolic filling pressure. Cardiac catheterization: equalization of diastolic pressures	Biopsy: fibrosis	Surgical pericardiectomy	Improvement on cyclophosphamide and prednisone	None
Schiavone *et al* Patient 2^1^	1985	USA	43/M	Nose, kidney	Edema, fevers, night sweats, hemoptysis and conjunctival erythema	Pericardial friction rub	EKG: diffuse STE, accelerated junctional rhythm and AV dissociation	TTE: small pericardial effusion	Not required/performed	Prednisone, cyclophosphamide	Symptomatic improvement on medical management	None
Schiavone *et al* Patient 3^1^	1985	USA	56/M	Lung	Productive cough, fever, night sweats	Transient pericardial friction rub, expiratory wheezing	EKG: atrial tachycardia with 2:1 conduction	TTE: small pericardial effusion	Not required/performed	Intravenous methylprednisolone and nitrogen mustard; digoxin and quinidine for heart block with conversion to sinus rhythm	Symptomatic improvement on medical management	None
Meryhew *et al*^2^	1988	USA	59/M	Lung, kidney	Dyspnea, fever, hemoptysis	Systolic ejection murmur	EKG normal, anemia, neutrophilia, thrombocytosis, elevated ESR, antinuclear antibody+	Chest CT: Large pericardial effusion. TTE: Large pericardial effusion with right atrial systolic and right ventricular diastolic collapse. Cardiac catheterization: equalization of diastolic intracardiac pressures	Pericardial window. Biopsy: acute inflammation, granulation tissue	Emergency pericardiectomy, intravenous methylprednisolone and cyclophosphamide	Improved symptomatically after hospital discharge on prednisone taper and cyclophosphamide but died suddenly at home 8 months later	None
Grant *et al* Patient 2^3^	1994	UK	45/M	Sinus	Heart failure	Engorged neck veins, peripheral edema, hepatomegaly, ascites, basal crackles in lungs	EKG: widespread nonspecific ST-T wave changes. Anemia, elevated ESR, c ANCA+	TTE: Small left ventricular cavity, no pericardial fluid or abnormality. Cardiac catheterization: constrictive pericarditis with equalization of diastolic pressures	Biopsy: Fibrosis	Prednisone, cyclophosphamide with mesna followed by pericardiectomy	Improvement on medical management	None
Grant *et al* Patient 3^3^	1994	UK	35/F	Sinus, kidney	Chest pain and increasing shortness of breath	Pale, puffy face and ankle swelling	Anemia, - c ANCA	TTE: moderately large pericardial effusion, abnormal right atrial movement, left ventricle small and vigorous with an estimated ejection fraction of 60%	Biopsy: Fibrinous hemorrhagic pericarditis	Pericardial fenestration followed by pericardiectomy	Did well and discharged home	None
Yildizer *et al*^4^	1996	Turkey	50/F	Sinus, lung, kidney	Cough, weakness, anorexia, pleuritic chest pain	Reduced breath sounds at right lung base	Anemia, elevated ESR, renal failure on dialysis	TTE: pericardial tamponade	Biopsy: necrotizing vasculitis	Prednisone, cyclophosphamide	Died during hospitalization	Not available
Florian *et al*^5^	2011	Belgium	38/M	Lung	Shortness of breath and position-related chest pain	No friction rub or murmur heard	EKG: diffuse T-wave flattening. Elevated ESR and CRP, c ANCA+	Chest CT: Mild cardiomegaly, discrete posterobasal pleural effusion, thickened pericardium without calcification. TTE: Thickened pericardium with circumferential, homogenous pericardial effusion (14 mm along LV wall), mitral valve: E inspiration 75 cm/sec, E expiration 97 cm/sec, 23% variation, E wave deceleration time 166 msec, normal BiV function. Cardiac MRI: real-time cine imaging showed minor septal flattening but no septal inversion no shift (argues against pericardial constriction), morphologic analysis by T1-weighted sequences showed normal myocardium and thickened pericardium (6 mm) with hyperintense circumferential pericardial effusion (up to 7 mm along the LV lateral wall). STIR imaging showed intense circumferential edema of both pericardial layers and limited subepicardial edema in inferolateral LV wall. Late post-gadolinium administration imaging showed strong enhancement of both pericardial layers, subtle subepicardial enhancement of inferolateral LV wall.	Not required/performed	Not reported	Not reported	Not reported
Somaliy *et al*^6^	2012	Saudi Arabia	34/M	Nasal sinus, lung, kidney	Chest pain and productive cough for 5 days, fever and arthralgia for 1 month	High jugular venous pulse, +Kussmaul sign, distant heart sounds	Neutrophilia, leukocytosis, elevated ESR, c ANCA+	Chest CT and TTE: Large pericardial effusion	Not required/performed	Intravenous prednisolone	Switched to oral prednisone with a prolonged taper; asymptomatic with resolution of pericardial effusion on 2-week follow-up	None
Horne *et al*^7^	2014	UK	42/F	Sinus, lung	Dyspnea, peripheral edema, orthopnea	Peripheral edema	EKG: normal; c ANCA+	TTE: good left ventrucular systolic function, diastolic septal bounce, increased respirophasic variaton of atrioventricular flows. Cardiac MRI: pericardial thickening (7 mm), and inspiratory septal flattening with no evidence of infiltrative/inflammatory myocardial disease	Biopsy: collagenous fibrous tissue with no evidence of inflammation or vasculitis	Surgical pericardiectomy	Improved symptomatically	None
Dewan *et al*^8^	2015	USA	57/M	None	Syncopal episode, frontal headache	None	Elevated ESR, p ANCA+	Chest CT: soft tissue attenuation around the coronary arteries, bypass grafts, pericardium. Cardiac MRI: enhancing soft tissue around the graft and coronary arteries with nodular appearance of pericardium	Biopsy: Dense scar tissue with mononuclear infiltrates: granulomatous capillaritis with leukocytoclasis and mononuclear infiltrate	Prednisone, rituximab	Improvement in soft tissue thickening around coronary arteries and pericardium at 3-year follow-up CT scan	None
Miyawaki *et al*^9^	2017	Japan	60/M	Lung, kidney	Fever, cough	Conjunctival hyperemia	Anemia, elevated CRP, c ANCA+	Chest CT: Thickened pericardium	Not required/performed	Methylprednisolone and cyclophosphamide	At 2- month follow-up: marked reduction in size of multicenter nodular pulmonary lesions, concentric soft tissue cuff around aortic arch and pericardial thickening	None
Parmar *et al*^10^	2019	USA	49/M	Sinus, Kidney	Dyspnea, chest pain	Saddle nose deformity, distant heart sounds, elevated jugular venous pulse, AV fistula bruit	EKG: electrical alternans. Anemia, elevated BUN and Cr, elevated ESR and CRP, p ANCA+	Chest CT: moderate pericardial effusion. TTE: pericardial effusion with tamponade	Pericardial window. Biopsy: acute inflammation, granulation tissue, fibrinopurulent exudate.	Pulse dose steroids with prolonged taper	Hospitalized within a month of discharge for arteriovenous fistula occlusion and sepsis/bacteremia; passed away secondary to cardiogenic shock and hypoxic respiratory failure during hospitalization.	Not available
Cleveland Clinic Patient 1	2020	USA	44/F	Sinus, lung, kidney	Positional chest pain, shortness of breath, fever	Friction rub	Leukocytosis, elevated ESR and CRP	Chest CT: Moderate-to-large sized pericardial effusion and left pleural effusion. TTE: moderate pericardial effusion without tamponade TEE: moderate pericardial effusion, no evidence of constriction	Not required	Prednisone 60 mg daily (with prolonged taper) and colchicine 0.6 mg twice daily	Multiple recurrences over the next 3 years, persistent after kidney transplant, requiring immunomodulatory therapy with anakinra with resolution of symptoms	Multiple
Cleveland Clinic Patient 2	2020	USA	63M	Sinus, ears	Shortness of breath, night sweats, dry cough	None	Leukocytosis, elevated ESR and CRP, elevated p-ANCA	Chest CT: large pericardial effusion, flattening of interventricular septum TTE: moderate organized circumferential pericardial effusion Cardiac MRI: Increased pericardial thickening of 2-3 mm with moderate circumferential enhancement of the pericardium on late gadolinium enhancement T1 sequence, increased signal on T2 edema weighted imaging	Pericardiocentesis, 400 mL of exudative effusion revealing acute and chronic inflammation	Colchicine 0.6 mg twice daily and ibuprofen 800 mg three times daily	Admitted with acute pericarditis after 1 month and managed with prednisone and cyclophosphamide. Currently on prednisone and rituximab with no reported recurrences.	None

*EKG*, Electrocardiogram; *F*, female; *LV*, left ventricular; *M*, male.

A comprehensive review of these cases revealed a male predominance (men, *n* = 10; women *n* = 3). The mean age of the patients was 48 years (range, 35-60 years). The common clinical presentation was cough with or without hemoptysis, fever, chest pain, and shortness of breath consistent with our experience of such patients. Physical examination findings of volume overload state were most frequently observed, whereas a pericardial friction rub and pericardial knock were less documented. Biochemical evaluation predominantly showed elevated ESR and CRP, as well as anemia, thrombocytosis, and neutrophilia associated with GPA. Patients 1 and 2 exhibited elevated inflammatory markers and leukocyte counts. Echocardiographic evidence of a new or worsening pericardial effusion was found to be present in most cases along with findings of tamponade and constrictive pathology in four and three patients, respectively. Two patients underwent advanced imaging with cardiac MRI, probably due to the recent surge in the use of this modality for the diagnosis of pericardial diseases. Patient 1 could not get an MRI in the setting of renal failure. In the case of patient 2, the diagnosis of acute pericarditis was evident on presentation; however, the cardiac MRI evaluation illustrating the findings associated with acute inflammatory pericarditis aided with the treatment response.

The 2015 European Society of Cardiology guidelines for the diagnosis and management of pericardial diseases define an episode of acute pericarditis as an inflammatory pericardial syndrome diagnosed with at least two of the following four criteria: (1) pericardial chest pain, (2) pericardial rubs, (3) new or widespread ST elevation or PR depression on the electrocardiogram, or (4) evidence of pericardial effusion (new or worsening) on imaging. The diagnosis is supported in the presence of elevated markers of inflammation (ESR, CRP, white blood cell count) and evidence of pericardial inflammation on imaging techniques (CT or MRI).^14^ Recurrent pericarditis (RP) is defined as recurrence of pericarditis after a documented first episode of acute pericarditis and a symptom-free interval of 4-6 weeks or longer.^14^ Acute pericarditis with transient constriction is stated to occur when the acute inflammation as well as constrictive features resolve with anti-inflammatory therapy.^14^

Transthoracic echocardiogram in suspected acute pericarditis provides confirmatory evidence of the diagnosis when a new or increasing pericardial effusion is found. As per our review, TTE was performed in all but two cases, in which the findings on a CT scan were found to be sufficient.^8^^,^^9^ Although not routinely used for the initial diagnosis of pericarditis, cardiac MRI is highly sensitive for the diagnosis of active pericarditis, and can be useful in cases with an otherwise uncertain diagnosis.^14^ Florian *et al*^5^ superbly described the cardiac MRI findings of pericardial involvement in GPA, suggesting that this imaging modality has a unique role to play, consistent with our observations with patient 2.

In this review, out of a total of 13, eight patients underwent pericardial biopsy, and evidence of acute inflammation and granuloma formation was found in five of these patients. In both patients 1 and 2, pericardial biopsy was avoided due to low diagnostic yield and patient comfort. A majority of the patients were treated with steroid therapy, often a combination of prednisone and cyclophosphamide. Surgical pericardiotomy was performed in patients with constrictive pericarditis. An interesting observation is that all patients improved clinically (except one case who died during hospitalization) with the interventions performed, with no reported recurrences during follow-up. Our experience is unique in patient 1, who presented with RP despite being on prolonged steroid therapy and subsequently responded to an immunomodulatory agent.

The pathophysiology of RP is postulated to be an amplified and self-sustained autoinflammatory and/or autoimmune response to exogenous or endogenous triggers. Anakinra, an interleukin-1 receptor antagonist, interferes with this self-sustained pathway and is among the emerging therapies for RP refractory to standard medical therapy to control symptoms and avoid the long-term effects of corticosteroids.^14^ Patient 1 developed steroid-refractory colchicine-resistant RP, which happened in the context of renal transplantation and did not respond to conventional anti-inflammatory therapy. Our patient showed a remarkable recovery with anakinra (Kineret, Sobi, Stockholm, Sweden). Currently approved by the U.S. Food and Drug Administration for the treatment of rheumatoid arthritis (off label for recurrent pericarditis), daily subcutaneous injections of anakinra at 1-2 mg/kg/day, up to 100 mg, for several months has been shown to lower the risk of recurrence, ED admissions, and hospitalizations and decreased use of corticosteroids in a multicenter observational cohort study.^14^^,^^15^ Anakinra is generally reserved for the most refractory pericarditis cases (especially if they are corticosteriod-dependent and colchicine-resistant) owing to high cost, daily subcutaneous injections and limited published data.^15^

## Conclusion

Cardiac involvement in GPA is found in advanced disease, with pericarditis being the most common clinical manifestation. Patients with a GPA flare can present with acute/RP as a manifestation of their flare. Pericarditis secondary to GPA, in the absence of constrictive physiology, usually responds to standard medical management. Steroid refractory pericarditis from GPA has not been previously reported, likely due to a paucity of long-term follow-up data. In patients with steroid-dependent/steroid-refractory pericarditis with multiple recurrences, anakinra seems promising for symptom control and to prevent recurrences and avoid the long-term effects of glucocorticoids.

## Supplementary Data

Video 1 Transthoracic echocardiogram with circumferential pericardial effusion on parasternal short-axis view.

Video 2 Transthoracic echocardiogram with circumferential pericardial effusion on apical four-chamber view.

Video 3 Transthoracic echocardiogram with apical four-chamber view with a respirometer showing no ventricular interdependence.

Video 4 On-treatment TTE videos of patient 1 showing no pericardial effusion or signs of pericardial constriction on parasternal short-axis view.

Video 5 On-treatment TTE videos of patient 1 showing no pericardial effusion or signs of pericardial constriction on parasternal four-chamber view.

Video 6 On-treatment TTE videos of patient 2 showing no pericardial effusion or signs of pericardial constriction on parasternal long-axis view.

Video 7 On-treatment TTE videos of patient 2 showing no pericardial effusion or signs of pericardial constriction on parasternal short-axis view.

Video 8 On-treatment TTE videos of patient 2 showing no pericardial effusion or signs of pericardial constriction on parasternal four-chamber view.
